# Prevalence of gastric cancer precursors in gastroscopy-screened adults by family history of gastric cancer and of cancers other than gastric

**DOI:** 10.1186/s12885-020-07612-8

**Published:** 2020-11-16

**Authors:** Rui Wu, Cheng Yang, Lin Ji, Zhi-Ning Fan, Yu-Wen Tao, Qiang Zhan

**Affiliations:** 1 Department of Gastroenterology, The Affiliated Wuxi People’s Hospital to Nanjing Medical University, 299 Qingyang Road, Wuxi, 214023 Jiangsu China; 2 Department of Digestive Endoscopy, The First Affiliated Hospital with Nanjing Medical University, 300 Guangzhou Road, Nanjing, 210029 Jiangsu China

**Keywords:** Family history, First-degree relative, Gastric precancerous lesions, Pathology

## Abstract

**Background:**

People are at a high risk of gastric cancer if their first-degree relatives suffered from atrophic gastritis (AG), intestinal metaplasia (IM), intraepithelial neoplasia (IEN), dysplasia (DYS), or gastric cancer (GC). This study was performed to analyse the association between FDR-GC and GC precursors.

**Methods:**

A cross-sectional study was performed to screen the prevalence of GC precursors from November 2016 to September 2019. A total of 1329 participants with FDR-GC, 193 participants with a family history of non-gastric cancer in FDRs (FDR-nGC), and 860 participants without a family history of cancer in FDRs (FDR-nC) were recruited in this study. The logistic regression model was used in this study.

**Results:**

The prevalence of normal, Non-AG, AG/IM, IEN/DYS, and GC was 31.91, 44.21, 13.81, 8.73, and 1.34%, respectively. The prevalence of IEN/DYS was higher in people with FDR-GC and FDR-nGC (FDR-GC: odds ratio (OR) = 1.655; 95%CI, 1.153–2.376; FDR-nGC: OR = 1.984; 95%CI, 1.122–3.506) than those with FDR-nC. The younger the age at which FDRs were diagnosed with GC, the more likely the participants were to develop AG/IM (*P*_trend_ = 0.019). The risk of precursors to GC was higher in participants whose FDR-GC was the mother than in those whose FDR-GC was the father or sibling (OR, non-AG: 1.312 vs. 1.007, 1.274; AG/IM: 1.430 vs. 1.296, 1.378; IEN/DYS: 1.988 vs. 1.573, 1.542). There was no statistically significant difference in non-AG (OR = 1.700; 95%CI, 0.940–3.074), AG/IM (OR = 1.291; 95%CI, 0.579–2.877), and IEN/DYS (OR = 1.265; 95%CI, 0.517–3.096) between participants with one or more FDR-GC.

**Conclusion:**

People with FDR-GC and FDR-nGC are at a high risk of IEN/DYS. When an FDR was diagnosed at a younger age, the risk of AG/IM was higher. The risk of GC precursors was higher in people whose FDR-GC was the mother.

## Background

Gastric cancer (GC) is associated with high incidence and mortality worldwide, especially in China, Japan, and Korea [1]. Gastric cancer has been a global health problem for a long time, with more than 1 million new cases and an estimated 783,000 deaths in 2018 all around the world [2]. Despite incidence and mortality of gastric cancer have decreased over the past 5 decades, gastric cancer remains the third leading cause of cancer-related death [3]. Meanwhile, the gastric cancer has three histological subtypes according to the Laurén classification (intestinal, diffuse and mixed type). Between 1989 and 2015, the relative median survival of non-metastatic intestinal and diffuse type gastric cancer improved from 22.8 to 27.6 months, and from 16.8 to 18.0 months, respectively [4]. The evidence was accumulated that the intestinal type of gastric cancer develops through a sequence of non-atrophic gastritis, atrophy, intestinal metaplasia, and dysplasia (intraepithelial neoplasia) to gastric cancer. However, the natural history of diffuse gastric cancer is unclear [5]. All these histological subtypes were relevant to our investigation.

Early detection of GC via screening endoscopy in asymptomatic patients enhances patient outcomes, especially in high-risk populations [6]. A Korean study [7] showed a 47% reduction in mortality after patients with GC underwent gastroendoscopy screening, and a 30% reduction was reported in Japan [8]. In China, a population-based study showed that people who underwent gastroendoscopy had a 28% reduced risk of mortality from GC [9]. However, compared to the large population in China, professionals and facilities are relatively limited, and we can now only screen individuals who are at a high risk of GC [10].

A family history of GC in first-degree relatives (FDRs) is one of the most important risk factors for GC [11, 12]. It has been reported that when the gastric lesions of FDRs change from a normal to precancerous status, and finally to GC, the incidence of GC in individuals increases significantly (7.7 × 10^− 5^ year^− 1^, 11.2 to 12.6 × 10^− 5^ year^− 1^, and 18.4 × 10^− 5^ year^− 1^, respectively) [13]. Meanwhile, some studies have reported that a family history of cancer in FDRs not only increases the risk of GC, but also increases the risk of gastric atrophy [14], which is an important precursor of GC [15]. In addition, patients with intestinal metaplasia have a significantly increased risk of GC [16]. However, there are few detailed reports of changes in gastric pathology in individuals with a family history of FDR-GC. Therefore, this study was conducted to examine in detail the gastric pathology of individuals with a family history of GC and provide a basis for screening of people at a high risk of GC.

## Methods

From November 2016 to September 2019, a cross-sectional study was performed to screen the prevalence of GC precursors. A total of 19,879 patients were given the serological tests, and 2382 patients underwent endoscopy and histopathological assessment.

The study protocol was reviewed and approved by the Ethics Committee of Wuxi People’s Hospital. All procedures performed in this study were in accordance with the ethical standards of the institutional and national research committee and the 1964 Declaration of Helsinki and its later amendments or comparable ethical standards. Each participant provided signed informed consent prior to enrolment.

### Study population

From November 2016 to November 2017, the first stage of the screening study at our digestive endoscopy centre was conducted by combining serological tests (pepsinogen and HP) with a family history of GC, and it was found that people with FDR-GCs had a higher gastroscopy screen-detected prevalence of GC [10]. In this stage, 7773 participants were serologically tested, and 872 underwent endoscopy and biopsy from 19,881 registered residents aged 40 to 69 years [17, 18] in seven communities in the Xinwu District of Wuxi City, Jiangsu Province, China. These seven communities were randomly selected from all 68 geographic communities in Xinwu District, and encompassed diverse areas.

**Fig. 1:**
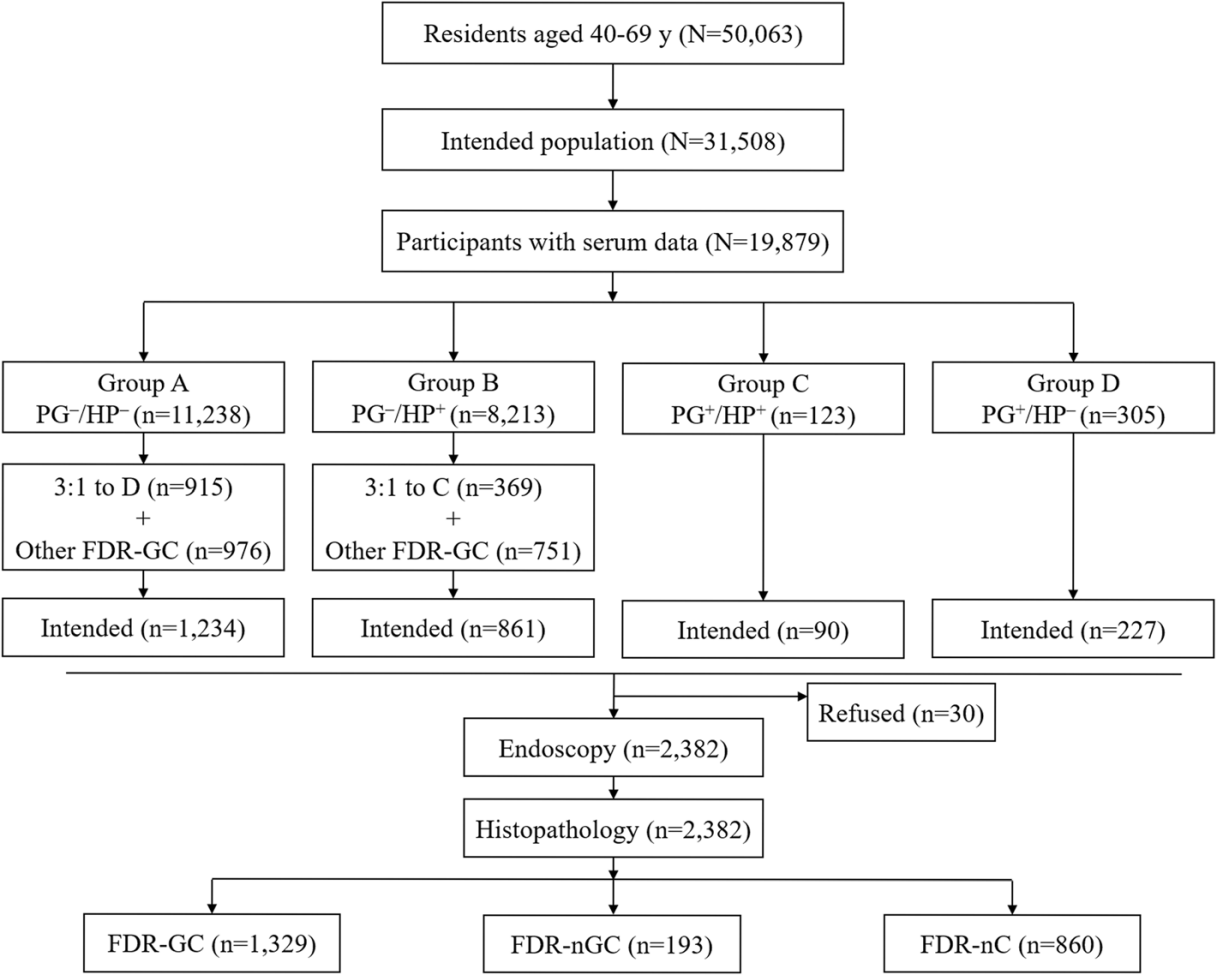
Process of inclusion for study population

From February 2018 to September 2019, the second stage of the screening study was performed using the same process, which was based on serological tests (pepsinogen and HP) and family history of GC. In these two stages, 50,063 registered residents aged 40 to 69 years from the same seven communities were recruited. Of these, 31,508 were willing to participate in the study, and participants with a history of developed cancer; gastric surgery, including endoscopic submucosal dissection or endoscopic mucosal resection; coagulopathy; and serious cardiopulmonary, liver, kidney, or psychiatric disorders; or those who failed to provide written informed consent were excluded from the study. Participants assured the researchers that they had not taken an acid suppressant or gastro-protective agent within the previous two weeks to prevent interference with levels of pepsinogen (PG), and also had not taken an antiplatelet drug such as aspirin to prevent bleeding during the endoscopic biopsy. Ultimately, 19,879 participants met the inclusion criteria, and fasting blood samples were collected for serological tests, including PGI, PGII, PGR, and anti-HP IgG. People who were positive for PG were all recruited for the study, and those who were negative for PG were recruited randomly. The PG^−^:PG^+^ ratio was 3:1 and it was stratified by H.pylori status. Further, this selection process resulted in intended participants comprising 74.1% of all participants in the PG^+^ group and 10.8% of those in the PG^−^ group. The remaining individuals with FDR-GC among 19,879 eligible participants were all recruited for the study as well (Fig. 1).

Ultimately, in two stages of the screening study, 1329 participants with FDR-GC, 193 participants with a family history of non-gastric cancer in first-degree relatives (FDR-nGC), and 860 participants without a family history of cancer in first-degree relatives (FDR-nC) underwent gastroendoscopy and histopathological assessment.

### Data collection

Information on sex, age, height (centimetres), weight (kilograms), family history of cancer, smoking (ever or never), drinking (ever or never), tea consumption (present or absent), intake of fruit and vegetables (<3times/week or ≥ 3times/week), and high-salt diet (present or absent) was collected from participants using self-assessment questionnaires. A positive family history was defined as a family history of cancer in the father, mother, sibling, or child of a participant. Participants with a positive family history were asked to provide cancer type and the age at diagnosis for each affected relative.

Fasting blood samples of the participants were collected for serological tests. PGI and PGII quantitative determination kits (Wuxi Jiangyuan Industrial Technology and Trade, Wuxi, Jiangsu, China) were used to examine the levels of PGI, PGII, and PGR via time-resolved fluoroimmunoassay in accordance with the manufacturer’s protocols. Atrophic serum PG (PG^+^) was defined as PGI < 60 ng/mL and PGR < 6.0, and the remaining population was defined as normal serum PG (PG^−^) in accordance with the manufacturer’s protocol. An HP antibody diagnostic test kit (Assure Tech, Hangzhou, Zhejiang, China) was provided to examine anti-HP IgG using a colloidal gold assay in accordance with the manufacturer’s manual. The details of the serological tests have been previously reported [19].

Twenty gastroenterologists each with more than 5 years of experience performed endoscopic procedures in 2382 participants. In every case, the gastric mucosa in the gastric antrum and gastric corpus was clipped for biopsy. Gastric mucosal biopsies were assessed in accordance with the standard criteria from the World Health Organization for the classification of GC and the updated Sydney System for grading and classification of gastritis by two expert gastrointestinal pathologists [20, 21]. Each participant who underwent gastroendoscopy and histopathological assessment was assigned a diagnosis of the gastric antrum, gastric corpus, and the whole stomach (including gastric antrum, gastric corpus, gastric angle, and gastric cardia). The diagnoses included normal, non-atrophic gastritis, atrophic gastritis/intestinal metaplasia, intraepithelial neoplasia/dysplasia, and GC.

### Statistical analysis

Analyses of demographic and clinical data were performed using SPSS software 21.0 version (IBM Corp, Armonk, NY, USA). We expressed the categorical variables as frequencies and percentages and the continuous variables as means ± standard deviations. Baseline characteristics were analysed using the chi-square test for categorical variables (sex, HP, family history, smoking, drinking, high-salt diet, tea, fruit/vegetables, and stage I and II) and analysis of variance for continuous variables (age, PGI/II, and BMI). A multinomial logistic regression model was used to analyse the pathological differences among FDR-GC, FDR-nGC, and FDR-nC. Pathological results were used as outcome variables (excluding the population diagnosed with GC (*N* = 32)), and the pathological results of the normal cases were used as the reference group. Odds ratios (ORs) and 95% confidence intervals (CIs) were calculated, and the level of statistical significance was set at *P* < 0.05.

## Results

### Characteristics of participants with different gastric pathologies

In this study, it was found that family history significantly affected gastric pathology (*P* = 0.019). Moreover, sex (*P* < 0.001), age (*P* < 0.001), HP (*P* < 0.001), PGR (*P* < 0.001), smoking (*P* < 0.001), drinking (*P* = 0.002), and tea consumption (*P* = 0.011) were closely related to gastric pathologies.

In addition, the gastric pathologies of the four groups in two stages are reported in Table 1. A total of 32 patients with GC and 208 patients with intraepithelial neoplasia/dysplasia was detected in these two stages.

**Table 1:** Characteristics of participants with different gastric pathologies

	Normal	Non-AG	AG/IM	IEN/DYS	GC	*P*
Overall	760(31.91%)	1053(44.21%)	329(13.81%)	208(8.73%)	32(1.34%)	
Family History						0.019
FDR-GC	394(29.65%)	599(45.07%)	190(14.30%)	126(9.48%)	20(1.50%)	
FDR-nGC	59(30.57%)	87(45.08%)	23(11.92%)	24(12.44%)	0(0.00%)	
FDR-nC	307(35.70%)	367(42.67%)	116(13.49%)	58(6.74%)	12(1.40%)	
Sex						< 0.001
Male	287(29.77%)	412(42.74%)	127(13.17%)	117(12.14%)	21(2.18%)	
Female	473(33.36%)	641(45.20%)	202(14.25%)	91(6.42%)	11(0.78%)	
Age	57.92 ± 7.70	58.62 ± 7.52	60.05 ± 7.16	61.19 ± 6.33	62.50 ± 5.70	< 0.001
HP						< 0.001
Positive	121(12.89%)	540(57.51%)	165(17.57%)	95(10.12%)	18(1.92%)	
Negative	639(44.28%)	513(35.55%)	164(11.37%)	113(7.83%)	14(0.97%)	
PGI/II	16.39 ± 7.41	12.78 ± 6.37	12.84 ± 7.13	13.68 ± 6.97	13.96 ± 11.56	< 0.001
Smoking						< 0.001
Ever	171(27.58%)	261(42.10%)	82(13.23%)	90(14.52%)	16(2.58%)	
Never	589(33.43%)	792(44.95%)	247(14.02%)	118(6.70%)	16(0.91%)	
Drinking						0.002
Ever	134(28.94%)	208(44.92%)	53(11.45%)	57(12.31%)	11(2.38%)	
Never	626(32.62%)	845(44.03%)	276(14.38%)	151(7.87%)	21(1.09%)	
High-Salt Diet						0.699
Present	606(31.50%)	859(44.65%)	262(13.62%)	169(8.78%)	28(1.46%)	
Absent	154(33.62%)	194(42.36%)	67(14.63%)	39(8.52%)	4(0.87%)	
Tea						0.011
Present	301(30.75%)	433(44.23%)	123(12.56%)	103(10.52%)	19(1.94%)	
Absent	459(32.72%)	620(44.19%)	206(14.68%)	105(7.48%)	13(0.93%)	
BMI	23.79 ± 2.88	24.51 ± 17.04	23.79 ± 2.97	23.81 ± 2.81	23.76 ± 3.08	0.687
Fruit/Vegetables						0.760
≥ 3Times/Week	487(32.08%)	669(44.07%)	209(13.77%)	136(8.96%)	17(1.12%)	
< 3Times/Week	273(31.60%)	384(44.44%)	120(13.89%)	72(8.33%)	15(1.74%)	
Stage I						< 0.001
Group A	158(38.16%)	154(37.20%)	63(15.22%)	35(8.45%)	4(0.97%)	
Group B	9(2.73%)	192(58.18%)	75(22.73%)	47(14.24%)	7(2.12%)	
Group C	3(7.50%)	23(57.50%)	9(22.50%)	5(12.50%)	0(0.00%)	
Group D	8(9.09%)	42(47.73%)	22(25.00%)	13(14.77%)	3(3.41%)	
Stage II						< 0.001
Group A	407(50.56%)	268(33.29%)	67(8.32%)	57(7.08%)	6(0.75%)	
Group B	95(18.13%)	304(58.02%)	74(14.12%)	41(7.82%)	10(1.91%)	
Group C	14(31.11%)	21(46.67%)	7(15.56%)	2(4.44%)	1(2.22%)	
Group D	66(48.53%)	49(36.03%)	12(8.82%)	8(5.88%)	1(0.74%)	

*Abbreviations*: *AG* atrophic gastritis, *IM* intestinal metaplasia, *IEN* intraepithelial neoplasia, *DYS* dysplasia, *GC* gastric cancer

Stage I, the study from November 2016 to November 2017 at our digestive endoscopy center; Stage I, the study from February 2018 to September 2019 at our digestive endoscopy center. Group A: people with PG^−^ and HP^−^; Group B: people with PG^−^ and HP^+^; Group C: people with PG^+^ and HP^+^; Group D: people with PG^+^ and HP^−^

### Pathological distribution in the gastric antrum, gastric corpus, and whole stomach

**Fig. 2:**
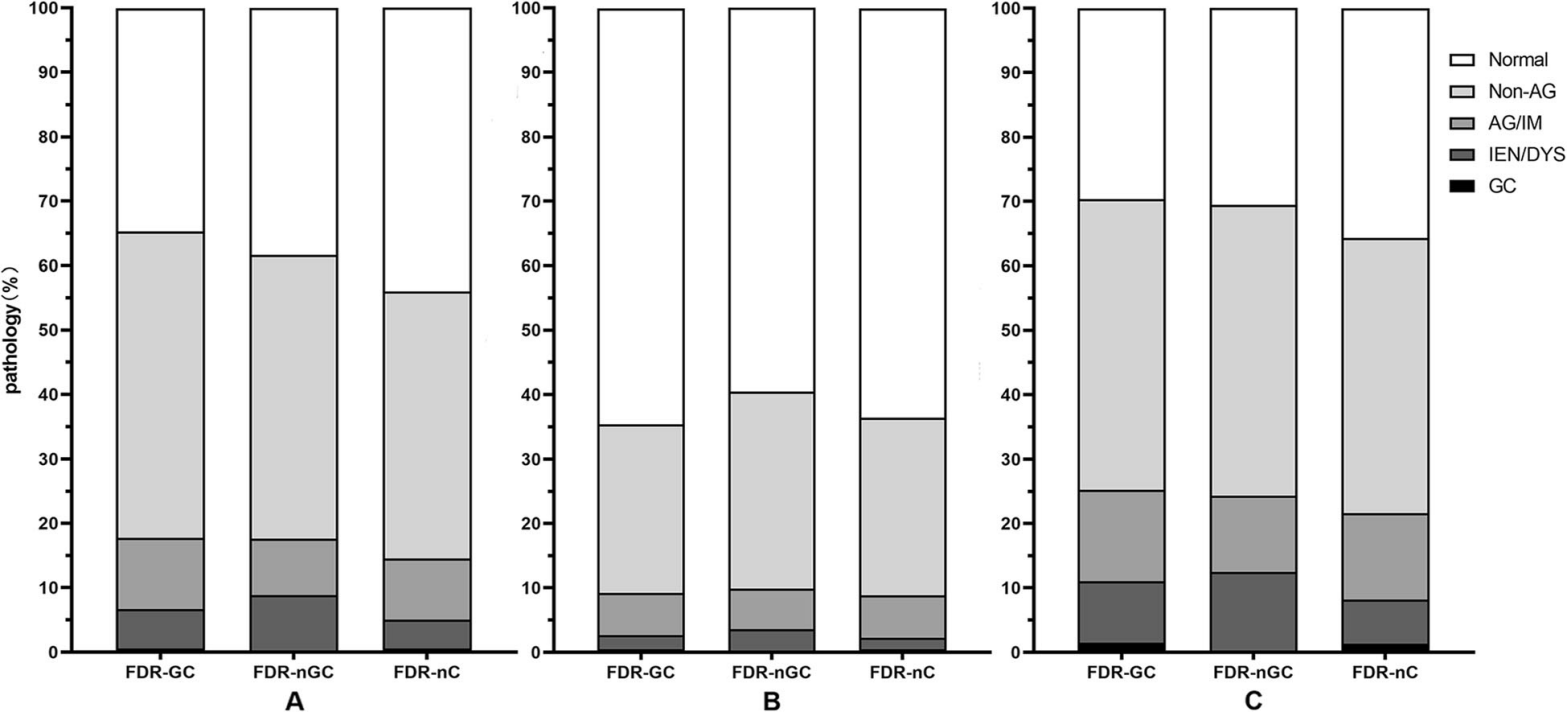
Pathological distribution in gastric antrum, gastric corpus and the whole stomach. **a**, gastric antrum; **b**, gastric corpus; **c**, the whole stomach. Abbreviations: AG, atrophic gastritis; IM, intestinal metaplasia; IEN, intraepithelial neoplasia; DYS, dysplasia; GC, gastric cancer

In the whole stomach (including gastric antrum, gastric corpus, gastric angle, and gastric cardia) of participants with FDR-GC, the proportion of atrophic gastritis/intestinal metaplasia (14.30% vs. 13.49%), intraepithelial neoplasia/dysplasia (9.48% vs. 6.74%), and GC (1.50% vs. 1.40%) was higher than that in participants with FDR-nC, while the proportion of normal and non-atrophic gastritis in participants with FDR-GC was lower (*P* = 0.021). Additionally, the proportion of intraepithelial neoplasia/dysplasia (12.44% vs. 6.74%) in participants with FDR-nGC was higher than that in participants with FDR-nC, while the proportion of normal, non-atrophic gastritis, and atrophic gastritis/intestinal metaplasia in participants with FDR-GC was lower (*P* = 0.027). However, none of the participants with FDR-nGC were diagnosed with GC. In addition, the pathological distribution of the gastric antrum was significantly different in patients with FDR-GC, FDR-nGC, and FDR-nC (*P* = 0.001), but there was no significant difference in the gastric corpus (*P* = 0.689) (Fig. 2**)**.

### Characteristics of participants with FDR-nC, FDR-nGC, and FDR-GC

There were significant differences in sex (*P* < 0.001), age (*P* < 0.001) and HP (*P* < 0.001) among participants with FDR-nC, FDR-nGC, and FDR-GC. At the same time, some lifestyle factors, such as smoking (*P* < 0.001), drinking (*P* < 0.001), and tea consumption (*P* < 0.001), also have significant differences for people in three groups **(**Table 2**)**.

**Table 2:** Characteristics of participants with FDR-nC, FDR-nGC, and FDR-GC

	FDR-nC	FDR-nGC	FDR-GC	*P*
Sex				< 0.001
Male	281(32.67%)	81(41.97%)	602(45.3%)	
Female	579(67.33%)	112(58.03%)	727(54.7%)	
Age	59.98 ± 7.35	60.81 ± 6.92	57.87 ± 7.5	< 0.001
HP				< 0.001
Positive	295(34.3%)	71(36.79%)	573(43.12%)	
Negative	565(65.7%)	122(63.21%)	756(56.88%)	
Smoking				< 0.001
Ever	170(19.77%)	47(24.35%)	403(30.32%)	
Never	690(80.23%)	146(75.65%)	926(69.68%)	
Drinking				< 0.001
Ever	121(14.07%)	39(20.21%)	303(22.8%)	
Never	739(85.93%)	154(79.79%)	1026(77.2%)	
High-Salt Diet				0.127
Present	676(78.6%)	157(81.35%)	1091(82.09%)	
Absent	184(21.4%)	36(18.65%)	238(17.91%)	
Tea				< 0.001
Present	303(35.23%)	84(43.52%)	592(44.54%)	
Absent	557(64.77%)	109(56.48%)	737(55.46%)	
BMI	23.65 ± 2.83	23.79 ± 2.99	24.45 ± 15.23	0.270
Fruit/Vegetables				0.313
≥ 3Times/Week	551(64.07%)	132(68.39%)	835(62.83%)	
< 3Times/Week	309(35.93%)	61(31.61%)	494(37.17%)	

### Gastric pathologies of people with a family history of cancer

People with a FDR-GC and FDR-nGC had higher prevalences of intraepithelial neoplasia/dysplasia compared to those with no family history of cancer in the whole stomach (9.48% vs. 6.74% OR = 1.655; 95% CI, 1.153–2.376 and 12.44% vs. 6.74% OR = 1.984; 95% CI, 1.122–3.506), respectively. Meanwhile, people with a FDR-GC had higher prevalence of atrophic gastritis/intestinal metaplasia compared to those with no family history of cancer, especially in the gastric antrum (11.06% vs. 9.53% OR = 1.426; 95% CI, 1.038–1.959). In addition, a family history of cancer did not significantly affect the gastric corpus. The OR value was adjusted by sex, age, HP, smoking, drinking, high-salt diet, and tea consumption. The findings for the gastric antrum, gastric corpus, and the whole stomach are reported in Table 3**and** Table 4.

**Table 3:** Crude odds ratios of whole stomach, gastric antrum and gastric corpus specific GC precursors associated with family history of gastric cancer and of cancers other than gastric

Exposure of interest	FDR-nC		FDR-GC			FDR-nGC		
Outcome	OR	N (%)	OR	95%CI	N (%)	OR	95%CI	N (%)
Gastric Antrum								
Non-AG	1	357 (41.51)	1.466	1.214–1.771	635 (47.78)	1.214	0.860–1.712	85 (44.04)
AG/IM	1	82 (9.53)	1.467	1.083–1.986	147 (11.06)	1.048	0.587–1.869	17 (8.81)
IEN/DYS	1	38 (4.42)	1.713	1.136–2.582	81 (6.09)	2.261	1.212–4.219	17 (8.81)
Gastric Corpus								
Non-AG	1	237 (27.56)	0.928	0.761–1.130	348 (26.19)	1.176	0.829–1.667	59 (30.57)
AG/IM	1	56 (6.51)	0.977	0.686–1.392	86 (6.47)	1.008	0.524–1.941	12 (6.22)
IEN/DYS	1	15 (1.74)	1.231	0.654–2.316	29 (2.18)	2.195	0.875–5.506	7 (3.63)
Whole Stomach								
Non-AG	1	367 (42.67)	1.272	1.043–1.550	599 (45.07)	1.234	0.857–1.774	87 (45.08)
AG/IM	1	116 (13.49)	1.276	0.969–1.680	190 (14.30)	1.032	0.609–1.748	23 (11.92)
IEN/DYS	1	58 (6.74)	1.693	1.199–2.390	126 (9.48)	2.153	1.241–3.737	24 (12.44)

Reference group: normal group

*Abbreviations*: *AG* atrophic gastritis, *IM* intestinal metaplasia, *IEN* intraepithelial neoplasia, *DYS* dysplasia, *GC* gastric cancer

**Table 4:** Adjusted odds ratios of whole stomach, gastric antrum and gastric corpus specific GC precursors associated with family history of gastric cancer and of cancers other than gastric

Exposure of interest	FDR-nC	FDR-GC		FDR-nGC	
Outcome	aOR	aOR	95%CI	aOR	95%CI
Gastric Antrum					
Non-AG	1	1.351	1.101–1.657	1.184	0.822–1.704
AG/IM	1	1.426	1.038–1.959	1.020	0.563–1.848
IEN/DYS	1	1.645	1.077–2.511	2.113	1.120–3.988
Gastric Corpus					
Non-AG	1	0.856	0.693–1.056	1.162	0.806–1.674
AG/IM	1	0.954	0.662–1.374	0.960	0.495–1.864
IEN/DYS	1	1.219	0.634–2.343	1.926	0.753–4.925
Whole Stomach					
Non-AG	1	1.171	0.946–1.451	1.208	0.824–1.773
AG/IM	1	1.284	0.961–1.716	1.008	0.586–1.734
IEN/DYS	1	1.655	1.153–2.376	1.984	1.122–3.506

Reference group: normal group

OR was adjusted by gender, age, HP, smoking, drinking, high-salt diet, and tea consumption

*Abbreviations*: *AG* atrophic gastritis, *IM* intestinal metaplasia, *IEN* intraepithelial neoplasia, *DYS* dysplasia, *GC* gastric cancer, *aOR* adjusted OR

### Influence of age at diagnosis of GC in FDRs on participants’ gastric pathologies

Among the 1309 participants with FDR-GC (20 patients diagnosed with GC were excluded), 1047 participants had a single FDR with only GC and no other cancer. Of the 1047 participants, 42 were unable to identify their age at diagnosis, while the remaining 1005 were included in the study. This avoided the confounding factors of type and number of people with a family history of cancer. The younger the FDRs were at the time of GC diagnosis, the more likely the participants were to develop atrophic gastritis/intestinal metaplasia (OR: < 50: 4.921; ≥50: 3.410; ≥60: 3.239; ≥70: 2.898; ≥80: 1; *P*_trend_ = 0.019), but this trend was not significant in people with non-atrophic gastritis (OR: < 50: 2.185; ≥50: 1.098; ≥60: 1.202; ≥70: 1.210; ≥80: 1; *P*_trend_ = 0.217) and intraepithelial neoplasia/dysplasia (OR: < 50: 2.372; ≥50: 1.039; ≥60: 1.517; ≥70: 1.578; ≥80: 1; *P*_trend_ = 0.823). The OR value was adjusted by sex, age, HP, smoking, drinking, high-salt diet, and tea consumption (Table 5).

**Table 5:** Influence of age at diagnosis with GC of first-degree relatives on participants’ gastric pathology

	N	OR	95%CI	OR^a^	95%CI
Non-AG					
< 50	40/73	1.905	0.894–4.057	2.185	0.965–4.950
≥ 50	102/230	1.026	0.587–1.793	1.098	0.598–2.017
≥ 60	159/353	1.092	0.643–1.855	1.202	0.674–2.145
≥ 70	114/262	0.969	0.561–1.675	1.210	0.666–2.198
≥ 80	41/87	1		1	
AG/IM					
< 50	11/73	3.667	1.136–11.838	4.921	1.439–16.832
≥ 50	40/230	2.817	1.080–7.345	3.410	1.256–9.257
≥ 60	54/353	2.596	1.018–6.621	3.239	1.222–8.582
≥ 70	35/262	2.083	0.797–5.447	2.898	1.065–7.887
≥ 80	6/87	1		1	
IEN/DYS					
< 50	7/73	1.556	0.485–4.992	2.372	0.692–8.130
≥ 50	17/230	0.798	0.320–1.990	1.039	0.398–2.711
≥ 60	36/353	1.154	0.500–2.661	1.517	0.631–3.650
≥ 70	29/262	1.151	0.489–2.709	1.578	0.639–3.893
≥ 80	9/87	1		1	

Reference group: normal group

The age of people in this study was between 26 and 88

^a^adjusted by gender, age, HP, smoking, drinking, high-salt diet, and tea consumption.

*Abbreviations: AG* atrophic gastritis, *IM* intestinal metaplasia, *IEN* intraepithelial neoplasia, *DYS* dysplasia, *GC* gastric cancer

### Influence of a FDR-GC who was the father, mother, or sibling on gastric pathology of the participants

Of the 1309 participants with FDR-GC (20 patients diagnosed with GC were excluded), 1047 participants had a single FDR with only GC and no other cancer (the FDR-GC of 669 participants was the father; for 235 participants, the FDR-GC was the mother; for 142 participants, the FDR-GC was the sibling; and for one participant, the FDR-GC was a child). A total of 1046 participants whose FDR-GC was the father, mother, or sibling were included in the study. Participants whose FDR-GC was the mother were more likely to suffer from non-atrophic gastritis (OR = 1.312; 95% CI, 0.902–1.907), atrophic gastritis/intestinal metaplasia (OR = 1.430; 95% CI, 0.872–2.345), and intraepithelial neoplasia/dysplasia (OR = 1.988; 95% CI, 1.116–3.542) than participants with FDR-nC, and the risk was higher than that for participants whose FDR-GC was the father or sibling (non-atrophic gastritis: 1.312 vs. 1.007, 1.274; atrophic gastritis/intestinal metaplasia: 1.430 vs. 1.296, 1.378; intraepithelial neoplasia/dysplasia: 1.988 vs. 1.573, 1.542). The OR value was adjusted by sex, age, HP, smoking, drinking, high-salt diet, and tea consumption (Table 6).

**Table 6:** Influence of participant with FDR-GC of father, mother, and sibling on gastric pathology, respectively

	FDR-GC			FDR-nC		FDR-GC		FDR-nC
	N	OR	95%CI	N	OR	OR^a^	95%CI	OR
Father								
Non-AG	292/669	1.136	0.901–1.433	367/848	1	1.007	0.778–1.305	1
AG/IM	99/669	1.219	0.885–1.678	116/848	1	1.296	0.916–1.834	1
IEN/DYS	63/669	1.551	1.043–2.307	58/848	1	1.573	1.024–2.417	1
Mother								
Non-AG	114/235	1.490	1.059–2.097	367/848	1	1.312	0.902–1.907	1
AG/IM	33/235	1.365	0.852–2.186	116/848	1	1.430	0.872–2.345	1
IEN/DYS	24/235	1.985	1.149–3.429	58/848	1	1.988	1.116–3.542	1
Sibling								
Non-AG	67/142	1.437	0.942–2.193	367/848	1	1.274	0.809–2.006	1
AG/IM	23/142	1.561	0.893–2.726	116/848	1	1.378	0.770–2.464	1
IEN/DYS	13/142	1.764	0.887–3.509	58/848	1	1.542	0.763–3.117	1

Reference group: normal group

^a^adjusted by gender, age, HP, smoking, drinking, high-salt diet, and tea consumption.

*Abbreviations*: *AG* atrophic gastritis, *IM* intestinal metaplasia, *IEN* intraepithelial neoplasia, *DYS* dysplasia, *GC* gastric cancer

### Influence of one or more FDR-GC on gastric pathology in the participants

Of the 1309 participants with FDR-GC (20 patients diagnosed with GC were excluded), 1047 participants who had only GC in a single FDR and 81 participants who had only GC in two or more FDRs were included in the study. There was no statistically significant difference in non-atrophic gastritis (OR = 1.700; 95% CI, 0.940–3.074), atrophic gastritis/intestinal metaplasia (OR = 1.291; 95% CI, 0.579–2.877), and intraepithelial neoplasia/dysplasia (OR = 1.265; 95% CI, 0.517–3.096) between participants with one or more FDR-GC. The OR value was adjusted by sex, age, HP, smoking, drinking, high-salt diet, and tea consumption (Table 7).

**Table 7:** Influence of participant with one or more FDR-GC on gastric pathology

	≥2 FDR-GC		Single FDR-GC
	OR	95%CI	OR
Non-AG	1.518	0.869–2.654	1
AG/IM	1.188	0.552–2.558	1
IEN/DYS	1.339	0.569–3.152	1
Non-AG^a^	1.700	0.940–3.074	1
AG/IM^a^	1.291	0.579–2.877	1
IEN/DYS^a^	1.265	0.517–3.096	1

Reference group: normal group

^a^adjusted by gender, age, HP, smoking, drinking, high-salt diet, and tea consumption.

*Abbreviations*: *AG* atrophic gastritis, *IM* intestinal metaplasia, *IEN* intraepithelial neoplasia, *DYS* dysplasia, *GC* gastric cancer

## Discussion

In our study, it was found that people with FDR-GC and FDR-nGC were related to the precursors of GC closely. In addition, the younger the age at which FDRs were diagnosed with GC, the more likely the participants were to suffer from atrophic gastritis/intestinal metaplasia. The risk of non-atrophic gastritis, atrophic gastritis/intestinal metaplasia, and intraepithelial neoplasia/dysplasia was also higher in participants with the FDR-GC who was the mother than in those with the FDR-GC who was the father or sibling.

Many studies have reported that GC has an underlying genetic predisposition [22, 23]. The risk of GC in migrants is similar to that in native people, but does not approach that in people in the first generation post-migration [24]. In a study of 4282 patients diagnosed with GC, Kwak [25] found that the average age at GC diagnosis in patients with paternal FDR-GC was significantly lower than that in those without FDR-GC (54.4 ± 10.4 vs. 58.1 ± 12.0, *P* < 0.001). Therefore, many studies have confirmed that people with FDR-GC are at a high risk of GC [26, 27]. In addition, people with precursors of GC such as atrophic gastritis and intestinal metaplasia on gastroendoscopy, had a significant risk of GC [15, 28]. However, the relationship between the family history of GC and GC precursors is rarely reported in detail. Therefore, this study was performed to screen for GC in high-risk groups.

In this study, it was found that people with FDR-GC were more likely to suffer from atrophic gastritis/intestinal metaplasia and intraepithelial neoplasia/dysplasia than people with FDR-nC, which is consistent with findings of previous studies [29, 30]. El-Omar et al. [31] reported that people in Scotland with FDR-GC had a higher prevalence of atrophic gastritis (34% vs. 5%) and intestinal metaplasia (19% vs. 12%) than people with FDR-nC. Meanwhile, we found that the gastric antrum was more prone to developing atrophic gastritis/intestinal metaplasia and intraepithelial neoplasia/dysplasia than the gastric corpus, and was more susceptible to FDR-GC. In addition, Gonzalez found that patients with atrophic gastritis and intestinal metaplasia are more likely to develop GC, especially in those with a family history of GC [32]. Therefore, people with FDR-GC require more intensive surveillance, with a particular focus on the gastric antrum.

In our study, none of the participants with FDR-nGC were diagnosed with GC. Song et al. found that except for a family history of breast cancer as a risk factor for GC, people with FDR-nGC did not have a significant increase in the risk of GC [14]. Foschi [33] and Dhillon [34] reported that the family history of non-gastric cancer was not an independent influential factor of GC, and our results were in accordance with these studies. Meanwhile, participants with FDR-nGC did not have an increased risk of non-atrophic gastritis and atrophic gastritis/intestinal metaplasia but were more likely to suffer from intraepithelial neoplasia/dysplasia than those with FDR-nC in our study. This may be the result of a combination of genetic and environmental factors [15]. Some studies reported that there was a tendency for the risks of GC to be above unity for a family history of cancer including cancers of the oesophagus, colorectum, liver, gallbladder, and pancreas, while none of the estimates were significant, and the relative risk of a family history of lung cancer was 1.5 for stomach cancer (95% CI, 1.0–2.3) [14, 35, 36]. Meanwhile, some genes play a role not only in GC but also in breast cancer [35, 37, 38]. Therefore, we must cautiously report that having FDR-nGC was a risk factor for intraepithelial neoplasia/dysplasia.

In this study, we found that the participants were associated with atrophic gastritis/intestinal metaplasia closely if their FDRs were diagnosed with GC at a younger age. In an American study [39], 19% of people under the age of 40 years who were diagnosed with GC had a positive family history, with the intestinal type of GC being the most common type [40], while western series reported a positive family history in less than 10% of individuals. Yu Bai reported that patients who were diagnosed with GC before the age of 35 years had a higher frequency of family history of GC (19%) with fewer alarming features [41]. These results suggest that the younger the age at diagnosis, the greater the influence family history may have on that individual. When an individual has an FDR diagnosed with GC at a young age, gastroendoscopy should be performed earlier in that individual.

In our study, participants whose FDR-GC was the mother had a higher risk of developing non-atrophic gastritis, atrophic gastritis/intestinal metaplasia, and intraepithelial neoplasia/dysplasia than those whose FDR-GC was the father or sibling. Palli reported that subjects were at a higher risk of GC with a maternal FDR-GC than with a paternal FDR-GC [42]. Zhou et al. suggested that a certain subtype of GC may be inherited in a female-influenced fashion [43]. However, Song et al. [13, 14] reported that compared with that of parents, the gastric pathology of siblings was more closely related to the gastric pathology of patients. Although studies of the impact of a sibling or parent FDR-GC on GC development have reported discrepant results, it was consistently reported that people with a maternal FDR-GC were more likely to develop GC than those with a paternal FDR-GC [44]. In addition, there was no statistically significant difference in non-atrophic gastritis, atrophic gastritis/intestinal metaplasia, and intraepithelial neoplasia/dysplasia between participants with one or more FDR-GC, and Bernini [45] reported that the number of FDR-GC did not affect the risk of GC.

The advantages of this study are obvious. Our study is novel in that few studies have reported the relationship between family history and GC precursors in detail. Additionally, our study had a large sample size with an asymptomatic population, which ensures the stability of the results. Furthermore, in people with or without a family history, 32 patients with GC were detected, most of whom were in early stages of GC (71.9%), which reflects the social benefits of this study. In addition, the structured interview-administered questionnaire concerning the patient’s family history was completed under the guidance of physicians, and Bravi et al. found that such a questionnaire was relatively reliable for data on family history of all cancers [46]. However, our study has many disadvantages. One of the limitations was that only 2382 of 3439 (69.26%) participants eventually underwent gastroendoscopy. This may have led to a potential bias. Analogously, in a Korean study, they found that people with or without a family history of GC were not all willing to undergo gastroendoscopy screening (39.2 and 32.3%, respectively) [47].

In further studies, we will increase the number of participants with FDR-GC to explore the detection rate of GC, especially early GC, in high-risk groups compared to the normal population. In addition, we believe that family history of first-degree relatives should be paid close attention to when screening for gastric cancer, so as to identify high-risk groups more accurately and improve the detection rate of precursors of GC and GC.

## Conclusion

People with FDR-GC are at a high risk of intraepithelial neoplasia/dysplasia. The younger the age at which FDRs were diagnosed with GC, the more likely the participants were to develop AG/IM. The risk of non-atrophic gastritis, atrophic gastritis/intestinal metaplasia, and intraepithelial neoplasia/dysplasia was also higher in participants with an FDR-GC who was the mother than in those with an FDR-GC who was the father or sibling. There was no statistically significant difference in the GC precursors between participants with one or more FDR-GC.

## Data Availability

Reasonable requests for data and materials will be considered and should be made in writing to the corresponding author.

## Abbreviations

AG: Atrophic gastritis
CI: Confidence interval
DYS: Dysplasia
FDR: First-degree relative
GC: Gastric cancer
IEN: Intraepithelial neoplasia
IM: Intestinal metaplasia
OR: Odds ratio
PG: Pepsinogen
